# Anterior colporrhaphy does not induce bladder outlet obstruction

**DOI:** 10.1007/s00192-012-1688-0

**Published:** 2012-02-08

**Authors:** M. M. E. Lakeman, R. A. Hakvoort, E. P. Van de Weijer, M. H. Emanuel, J. P. W. R. Roovers

**Affiliations:** 1 Department of Obstetrics and Gynaecology, Academic Medical Centre, Room H4-205, PO Box 22700, 1105 DE Amsterdam, The Netherlands; 2 Department of Obstetrics and Gynaecology, Spaarne Hospital, Hoofddorp, The Netherlands; 3 Department of Urology, Spaarne Hospital, Hoofddorp, The Netherlands

**Keywords:** Bladder outlet obstruction, Prolapse surgery, Urinary retention, Urodynamics

## Abstract

**Introduction and hypothesis:**

We aimed to evaluate if anterior colporrhaphy causes incomplete voiding due to bladder outlet obstruction.

**Methods:**

Women scheduled for anterior colporrhaphy were asked to undergo multichannel urodynamic investigation before surgery and the first postoperative day. Bladder outlet obstruction was assessed using the Blaivas–Groutz voiding nomogram. Maximum flow rate, detrusor pressure and residual volume were compared between pre- and postoperative measurements and between women with and without an abnormal post-void residual volume (PVR; volume exceeding 150 ml).

**Results:**

Seventeen women participated. One woman who was unobstructed before surgery was obstructed after surgery. Overall, detrusor pressure and maximum flow rate before and after surgery did not differ. After surgery, six women had an abnormal PVR, one was unable to void, four were mildly obstructed and one moderately obstructed.

**Conclusion:**

Urodynamic investigation the first day after anterior colporrhaphy did not show that anterior colporrhaphy induces bladder outlet obstruction. The explanation for postoperative urinary retention can therefore also lie in non-anatomical causes such as postoperative pain and psychological factors.

## Introduction

Vaginal prolapse surgery is intended to restore normal pelvic floor function by correcting anatomical abnormalities. One of the most common complications directly related to prolapse surgery is the occurrence of incomplete emptying of the bladder [1]. Whereas the optimal management is assessed in several studies, the underlying pathophysiology of voiding difficulties after vaginal prolapse surgery is poorly understood [2].

Several hypotheses can be raised like impairment of bladder function and pelvic floor relaxation due to postoperative pain and anxiety [3]. Other frequently raised hypotheses have been urethral obstruction by oedema or hematoma formation and surgical damage to the innervation of the bladder [4]. As incomplete voiding is generally short lasting, it is questionable whether innervation damage plays an important role and thus obstruction related to oedema formation is a more acceptable explanation [5]. Until now, no studies have been undertaken to objectify the occurrence of bladder outlet obstruction following anterior colporrhaphy.

Previous studies have indicated that a combination of clinical parameters and urodynamic findings is the best way to define bladder outlet obstruction [6]. During urodynamics, flow rate and detrusor pressure are measured simultaneously and together they provide insight in whether obstruction is present as indicated by a relatively low flow rate related to the measured detrusor pressure [3, 4, 6, 7]. Therefore, urodynamic studies were performed on patients before and after surgery in a prospective observational study to assess if anterior colporrhaphy is a risk factor for postoperative bladder outlet obstruction.

## Materials and methods

A prospective study was performed in the Spaarne Hospital, Hoofddorp, the Netherlands. Women aged 18 years and older and who were scheduled for anterior colporrhaphy were informed about the study and asked to participate. Anterior repair could be combined with posterior repair and/or vaginal hysterectomy, sacrospinous ligament fixation or Manchester repair. Patients who were diagnosed with any neurological or anxiety disorder for which they underwent professional treatment and patients undergoing concomitant incontinence surgery were excluded.

The study was approved by the regional medical ethics committee (VU Academic Medical Centre, Amsterdam) and by the institutional medical ethical committee. After informed consent was obtained, patients underwent multichannel urodynamic investigation within 2 weeks before surgery and between 12 and 24 h after surgery.

During the study period, the participants completed a standardised urogynaecologic interview. Baseline characteristics and procedures performed were collected from all women.

### Urodynamic investigations

Before urodynamics, urine analysis and culture were performed to rule out significant bacteriuria (defined as more than 10^5^ colony-forming units) and cystitis (defined as bacteriuria with at least one of the following additional complaints: lower abdominal pain, dysuria or fever). In case of urinary tract infection, patients were excluded to minimise the chance of urodynamic artefacts and interference by urinary tract infection. Prior to the pressure flow studies, patients were asked to empty their bladders; after which, the bladder was drained by a hydrophilic-coated transurethral catheterisation (SpeediCath®, Amersfoort, the Netherlands).

Subsequently, pressure flow studies were performed using a MMS UD 2000 device (Medical Measurement Systems MMS, Enschede, Netherlands) with a water-filled MediPlus 5716 double-lumen cystometry catheter and a water-filled MediPlus 5415 rectal pressure balloon catheter.

Filling of the bladder occurred with saline at body temperature with a speed of 50 ml/min up to the moment patients experienced a strong desire to void or either filling continued up to a maximum volume equalling the functional bladder capacity which was defined as the largest voided volume in a 24-h voiding a sitting position with the 7F catheter in place. The post-void residual volume (PVR) was calculated by bladder catheterisation. Patients with a post-void bladder volume exceeding 150 ml were diagnosed as having an abnormal PVR.

### Surgery

Anterior colporrhaphy was performed using a midline incision of the vaginal epithelium, and the bladder was sharply dissected from the vaginal wall. The pubocervical fascia was plicated in the midline with absorbable Vicryl® 2–0 interrupted sutures (Ethicon Inc, Somerville, NJ, USA). The surplus of vaginal epithelium was removed, and the epithelium was closed with running absorbable interlocking Vicryl 2–0.

All procedures were performed in the same hospital and were performed by two gynaecologists with a special interest in urogynaecology. All procedures were performed under spinal analgesia. Patients received postoperative prophylaxis for deep vein thrombosis and a single dose of intravenous prophylactic antibiotics during surgery. A 14 French Foley indwelling catheter with a 5-ml balloon was used to drain the bladder after surgery. This catheter was removed within 24 h on the morning of the first day after surgery.

### Postoperative care

Postoperative care was standardised for all patients. A vaginal gauze was inserted directly after surgery. The catheter and gauze were removed on the morning of the first postoperative day. After the first attempt to void, patients underwent catheterisation of the bladder and subsequently pressure flow studies were performed (see “Urodynamic investigations” section). Patients with a PVR above 150 ml received additional transurethral indwelling catheterisation for the duration of 3 days.

### Outcome measurements

The primary outcome was the presence and extent of obstruction before and after surgery as defined by the Blaivas and Groutz nomogram [6, 8]. In this nomogram, the maximum flow rate (*Q*_max_) is plotted in relation to the maximum detrusor pressure (*P*_detmax_) (obtained from the pressure flow study). Four categories of obstruction have been defined: no, mild, moderate and severe obstruction. The boundaries of the four categories are as follows:

- Between no obstruction and mild obstruction: a line with slope 1.0 and intercept 7 cm H2O
- Between mild and moderate obstruction a horizontal line at *P*_detmax_ of 57 cm H2O
- Between moderate and severe obstruction a horizontal line at *P*_detmax_ of 107 cm H2O

Secondary outcomes were differences in maximum flow rate, maximum detrusor pressure, maximum detrusor pressure during maximum flow rate and residual volume pre- and postoperatively in the total group and between women with and without abnormal PVR. A subanalysis was performed in women with abnormal PVR to compare the pre- and postoperative measurements.

### Statistical analysis

Data were analysed in SPSS version 18.0. Continuous variables were analysed using the Wilcoxon’s test for dependent data (i.e. differences between pre- and postoperative measurements) and a Mann–Whitney test for independent data. For categorical variables, Fisher exact or chi-square test was used.

## Results

During the study period, 17 women underwent urodynamic investigation before and after anterior colporrhaphy was performed. Baseline characteristics and concomitantly performed procedures are summarised in Table 1.

**Table 1:** Patient characteristics, performed procedures and operative characteristics of the women who underwent pre- and postoperative pressure flow studies

	*N* = 17
Patient characteristics	
Age (years)	61.2 (38.3–76.8)
Parity (*n*)	3 (2–8)
BMI (kg/m^2^)	26.7 (22.4–32.2)
Previous gynaecological procedures	
Hysterectomy	2 (12)
POP-Q	
Ba	0 (−0.5–1.5)
Bp	−2.6 (−3.0–0.0)
C	−2.4 (−5.0–0.0)
Performed procedure	
Anterior colporraphy only (AC)	11 (65)
AC + sacrospinous ligament fixation	1 (6)
AC + posterior colporrhaphy (PC)	3 (18)
AC + Manchester Fothergill	1 (6)
AC + Manchester Fothergill + PC	1 (6)
Operative characteristics	
Duration of surgery (min)	32.0 (25.0–78.0)
Surgical complications	0 (0)
Postoperative complications	0 (0)

Values are numbers (percentage) or median (range)

**Fig. 1:**
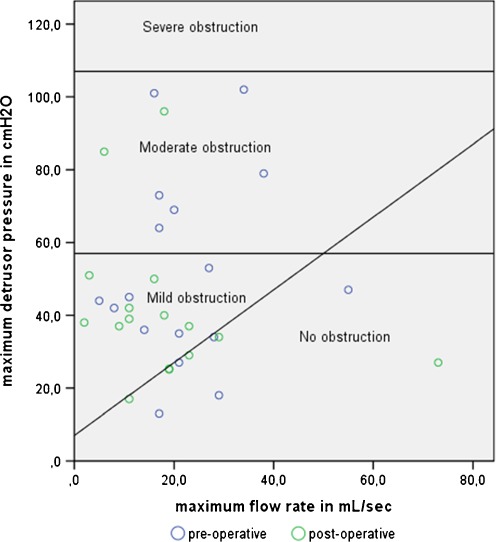
Blaivas and Groutz nomogram. Distribution of the maximum flow rate by maximum detrusor pressure before and after vaginal prolapse surgery was performed

Figure 1 shows the Blaivas and Groutz nomogram before and after surgery. The presence and degree of pre- and postoperative bladder outlet obstruction are summarised in Table 2. Overall, before surgery, five women were unobstructed, one of them had de novo mild bladder outlet obstruction; she underwent anterior colporrhaphy and had a residual volume after voiding of 121 ml. Twelve women were obstructed preoperatively, two of them were no longer obstructed after surgery. From the ten women who had pre- and postoperative bladder outlet obstruction, one woman increased in degree of obstruction from mild to moderate. She underwent anterior and posterior colporrhaphy and had a residual volume of 441 ml.

**Table 2:** Classification of obstruction according to the Blaivas and Groutz nomogram before and after surgery was performed

			Postoperative situation			
			No obstruction	Mild obstruction	Moderate obstruction	Severe obstruction
Preoperative situation	No obstruction	5 (29%)	4	1	0	0
	Mild obstruction	6 (35%)	0	5	1	0
	Moderate obstruction	6^a^ (35%)	2	2	1	0
	Severe obstruction	0 (0%)	0	0	0	0
Total		17	6	8	2	0

^a^One patient was not able to void postoperatively

Table 3 shows the urodynamic findings before and after surgery. No statistical significant differences were found between the pre- and postoperative measurements.

**Table 3:** Comparison of the median maximum flow rate (*Q*_max_), maximum detrusor pressure (*P*_detmax_), maximum detrusor pressure during maximum flow rate (*P*_detQmax_), residual volume and flow time as obtained by pressure flow studies before and after surgery

	Before surgery, *n* = 17	After surgery, *N* = 16^a^	*P* ^b^
Voided volume (in mL)	364.0 (3.0–712.0)	338.0 (7.0–588.0)	0.38
*Q*_max_ (in mL/s)	20.0 (5.0–55.0)	17.0 (0.0–73.0)	0.36
*P*_detQmax_ (in cm H2O)	22.0 (0.0–73.0)	26.0 (0.0–69.0)	0.80
*P*_detmax_ (in cm H2O)	44.0 (13.0–102.0)	37.5 (17.0–96.0)	0.53
Residual volume (in mL)	10.0 (0.0–707.0)	66.0 (0.0–487.0)	0.78
Flow time (in s)	49.5 (28.0–222.0)	58.0 (9.0–255.0)	0.65

Values are median (range)

^a^One patient was unable to void during pressure flow studies after surgery

^b^As calculated using Wilcoxon signed-ranks test

Postoperatively, six women had an abnormal PVR (range, 172–487 ml). All of these six patients received a transurethral indwelling catheter which was removed after 3 days. All patients showed a residual volume under our definition of abnormal residual volume of 150 ml after removal of this catheter. Of the six women with an abnormal PVR, one could not void at all, four women were classified as mildly obstructed and one woman as moderately obstructed according to the Blaivas and Groutz nomogram. One of these six women also had an abnormal PVR before surgery, the others not. Two of the six women underwent anterior and posterior colporrhaphy, the others anterior colporrhaphy only. Table 4 shows the postoperative urodynamic findings in these women compared to women with a normal PVR. Women with abnormal PVR had a statistically significant lower maximum flow rate. No difference was found in maximum detrusor pressure. When performing a subanalysis comparing urodynamics before and after surgery among women with abnormal PVR, no statistically significant differences were found in maximum detrusor pressure, maximum detrusor pressure at maximum flow and maximum flow rate (data not shown).

**Table 4:** Comparison of postoperative median maximum flow rate (*Q*_max_), maximum detrusor pressure (*P*_detmax_), maximum detrusor pressure during maximum flow rate (*P*_detQmax_), residual volume and flow time between women with and without abnormal PVR

	Normal PVR, *n* = 11	Abnormal PVR, *N* = 6^a^	*P* ^b^
Voided volume	406.0 (116.0–588.0)	98.0 (7.0–206.0)	0.01
*Q*_max_ (in mL/s)	19.0 (11.0–73.0)	6.0 (2.0–11.0)	0.00
*P*_detQmax_ (in cm H2O)	32.0 (10.0–45.0)	14.0 (0.0–69.0)	0.32
*P*_detmax_ (in cm H2O)	34.0 (17.0–96.0)	39.0 (37.0–85.0)	0.15
Residual volume (in mL)	0.0 (0.0–141.0)	392.0 (305.0–707)	0.00
Flow time (in s)	54.0 (35.0–108.0)	82.0 (9.0–255.0)	0.74

^a^One patient was unable to void during pressure flow studies after surgery

^b^As calculated using Mann–Whitney *U* test

## Discussion

Our study was intended to explore if anterior colporrhaphy causes bladder outlet obstruction. Using urodynamic investigations shortly before surgery and on the first day after surgery, we could not reveal any difference in the presence of bladder outlet obstruction, the degree of obstruction, detrusor pressure and maximum flow rate. De novo obstruction after anterior colporrhaphy was only found in one woman, questioning the contribution of bladder outlet obstruction to the development of incomplete voiding following anterior colporrhaphy.

Before further interpreting the data, some issues need to be discussed. First is the relatively low number of patients included. For the diagnosis of obstruction, generally a *Q*_max_ <12 ml/s is required [7]. In the preoperative situation, median maximum flow rate was 20 ml/s; therefore, we needed to be able show an 8-ml/s difference to be able to show if prolapse surgery caused bladder outlet obstruction. According to a post hoc analysis, this study would have 80% power to pick up a mean difference in maximum flow rate of >7.7 ml/s between pre- and postoperative situation. Therefore, we are confident that we were able to pick up relevant differences with this small sample size. Furthermore, since no previous studies are performed evaluating bladder outlet obstruction, the first intention of our study was to explore if we could find any evidence that the inability to void on the first postoperative day was caused by bladder outlet obstruction possibly caused by oedema or hematoma formation. Even with our limited sample size, we could not reveal any indication that this was the case; therefore, we think it is unethical to expose more women to this investigation.

Second, one can argue about the timing of removal of the catheter and the consequent assessment of voiding parameters by postoperative pressure flow study. We decided to remove the catheter within the first day after surgery because several studies have shown benefits regarding UTI risk and catheterisation duration of this regimen [9–11]. This is therefore the timing at which incomplete voiding is most commonly identified and therefore the most clinically relevant timing when intending to study if bladder outlet obstruction plays a role in the development of this complication.

All included women underwent anterior colporraphy. Concomitant surgery included suspension techniques for uterine descent (two patients), posterior colporraphy (three patients) and a combination of both in one patient. Suspension techniques and elevation of the apical portion of the vagina have been reported to be a risk factor for abnormal PVR [12]. The most likely explanation for this finding is extra elevation of the bladder outlet and therefore obstruction. From our data, we could not confirm that obstruction according to the Blaivas and Groutz nomogram increased in the three patients with concomitant uterine suspension. Two studies have shown a risk increase with the performance of posterior compartment surgery. As this type of surgery has no anatomical relationship with the bladder, it has been hypothesised that pain and a disturbed relaxation of the pelvic floor might be a contributing factor in these cases [13, 14]. In our study in one of the three patients with concomitant posterior repair obstruction according to the Blaivas and Groutz nomogram increased from mild to moderate, one patient could not void after surgery and the last patient remained unaltered. This does not exclude that posterior colporraphy might increase the risk of abnormal PVR due to the previously mentioned hypothesis. However, due to the small numbers, no definite conclusions can be drawn.

We chose to use pressure flow studies to assess bladder outlet obstruction since this is the best defined way to assess obstruction [4]. One might argue that video urodynamics has the advantage of also localising a possible obstruction; however, other studies have shown that using cutoff points in pressure flow studies compares favourably to video urodynamics [4, 15]. Imaging techniques such as MRI have the advantage of visualisation of the obstruction; however, these techniques cannot be combined with flow studies and are therefore not able to assess the functional component which is most relevant when assessing causes of abnormal PVR [5].

Our main outcome was the presence and extent of obstruction as defined by the Blaivas and Groutz nomogram [6]. The original Blaivas and Groutz nomogram uses maximum flow rate obtained by free flow because in their series a significantly higher flow rate was observed in the same patient without the presence of a catheter [6]. We chose not to analyse maximum flow rate as obtained by free flow as such measurement implicates that the measured values of maximum flow rate and detrusor pressure are based on two separate and potentially different voids as one originates from a void with a catheter and one without. Furthermore, we wanted to minimise the burden for the patients. However, by measuring maximum flow rate with the catheter present, it is possible that we subsequently obtained a relatively low maximum flow rate which might explain part of the high rate of obstruction we found before and after surgery using the Blaivas and Groutz nomogram.

Recently, Massolt et al. also suggested that the Blaivas and Groutz nomogram might overestimate the proportion of patients with bladder outlet obstruction [8]. When using other cutoff points, we would probably have found less obstructed women [3, 4, 7, 16]. However, when using these cutoff point studies, one has to realise that only patients with clinically predefined anatomical obstruction were included in these studies, whereas women with functional bladder neck obstruction, e.g. from surgery, were not included in any of these studies [4]. Therefore, the Blaivas and Groutz nomogram might still be the most informative since it graphically shows the difference in flow rates and detrusor pressure which also enables us to see smaller differences between the pre- and postoperative situation.

The main goal of this study was to investigate if anterior colporrhaphy causes bladder outlet obstruction which is hypothesised to be due to urethral elevation or either by suburethral hematoma and/or oedema formation. Using the Blaivas and Groutz nomogram, only one woman appeared to develop obstruction postoperatively, and she was only mildly obstructed after surgery. Also, when looking at the detrusor pressure, no evidence for obstruction could be found since we did not see a rise in detrusor pressure during maximum flow rate. This does not exclude that oedema formation or elevation of the bladder neck might be present; however, our data show that if oedema formation is present, it does not seem to introduce obstruction to the bladder outlet more than the situation before surgery.

In addition, damage to the innervation of the bladder is previously hypothesised as a possible cause for incomplete voiding after prolapse surgery [14]. This would result in a decrease in maximum detrusor pressure and detrusor pressure during maximum flow rate. In women with abnormal PVR, we did observe a trend towards a lower detrusor pressure during maximum flow rate and observed a decreased flow rate. This might indicate that innervation damage may play a role in the development of abnormal PVR. However, an argument against this hypothesis is that within the group of women with abnormal PVR, we could not show a decrease in detrusor pressure during maximum flow rate when comparing detrusor pressure pre- and postoperatively.

Considering the limited evidence that has been provided to support the role of innervation damage on incomplete bladder emptying, and because of the lack of evidence for obstruction as a causal factor for incomplete bladder emptying, we think the underlying pathophysiology of voiding difficulties after prolapse surgery should not only be sought in mechanical causes. It is possible that other factors like pain and postoperative anxiety will contribute to this complication [13, 14]. Support for a possible role of postoperative anxiety came from three earlier randomised studies which all showed a reduction of the postoperative incidence of abnormal PVR after urogynaecological surgery with the administration of alpha blocking agents [17–19]. Further, previous studies have also shown that bladder function impairment could also be explained by psychological inhibition due to the clinical environment in which patients are requested to void postoperatively [17–20]. Therefore, we think that future research should focus more on the origin and treatment of these non-mechanical causes as with the present study and current literature most evidence points towards that direction.

## Concluding message

Urodynamic investigation on the first day after anterior colporrhaphy shows that anterior colporrhaphy carries a low risk of inducing bladder outlet obstruction. The explanation for postoperative development of abnormal PVR should therefore not only be sought in the effects of surgery on the bladder neck and urethra but also involves other non-anatomical explanations such as anxiety, pain and other psychological factors. These possible candidates should be evaluated in order to decrease the prevalence of abnormal PVR and optimise its treatment.
